# Supplementary material for: NME3 binds to phosphatidic acid and mediates PLD6-induced mitochondrial tethering
Source: J Cell Biol. 2023 Aug 16;222(10):e202301091. doi: 10.1083/jcb.202301091 (PMC10432850; doi:10.1083/jcb.202301091)
Supplement: Table S1 — lists the plasmids used in this study. [file JCB_202301091_TableS1.docx]

| **Supplementary tables** | |  |
| --- | --- | --- |
| **Table S1** |  |  |
| List of plasmids used in this study. | |  |
|  |  |  |
| Plasmids | Vector | Source |
| PLD6-mCherry | mCherry- N1 | In this study |
| NME3 ^WT^-GFP | EGFP-N1 | In this study |
| NME3 ^H135Q^-GFP | EGFP-N1 | In this study |
| NME3 ^E40/46D^-GFP | EGFP-N1 | In this study |
| NME3^F9/13A^-GFP | EGFP-N1 | In this study |
| NME3 ^ΔN^-GFP | EGFP-N1 | In this study |
| N17-GFP | EGFP-N1 | In this study |
| N17^F9/A^-GFP | EGFP-N1 | In this study |
| N17^F9/13A^-GFP | EGFP-N1 | In this study |
| NME3^WT^-His | pET30a | In this study |
| NME3 ^E40/46D^-His | pET30a | In this study |
| NME3 ^F9/13A^ -His | pET30a | In this study |
| NME3 ^ΔN^ –His | pET30a | In this study |
| optoPLD^WT^ (mito-targeted) | mCherry-CRY2 | In this study |
| optoPLD^H170A^ (mito-targeted) | mCherry-CRY2 | In this study |
| Neon-FKBP-DNNME3^WT^ | PCMV-Neon-FKBP | In this study |
| Neon-FKBP-DNNME3^H135Q^ | PCMV-Neon-FKBP | In this study |
| Neon-FKBP-DNNME3^E40/46D^ | PCMV-Neon-FKBP | In this study |
| NME3 ^WT^-HA | pcDNA.3 | In this study |
| NME3 ^E40/46D^-HA | pcDNA.3 | In this study |
| ALPS-NME3-GFP | EGFP-N1 | In this study |
| Mito-BFP | pAcGFP-N1 | Addgene |
| TOM20-GFP | EGFP-N1 | Addgene |
| TOM20-mCherry | mCherry-N1 | Addgene |
