# Supplementary material for: NME3 binds to phosphatidic acid and mediates PLD6-induced mitochondrial tethering
Source: J Cell Biol. 2023 Aug 16;222(10):e202301091. doi: 10.1083/jcb.202301091 (PMC10432850; doi:10.1083/jcb.202301091)
Supplement: Table S2 — lists the antibodies used in this study. [file JCB_202301091_TableS2.docx]

| **Table S2** |  |  |
| --- | --- | --- |
| List of antibodies used in this study. | |  |
| Targeted proteins | Sources | Dilution factor |
| 6xHis | Santa Cruz, sc-8036 | 1:1000 |
| GFP | A kind gift from Prof. Fang-Jen Lee | 1:1000 |
| alpha-tubulin | Sigma, T6074 | 1:5000 |
| beta-tubulin | Sigma, T4026 | 1:2000 |
| PLD6 | Abcam, ab237612 | 1:2500 |
| TOM20 | Santa Cruz, sc-17764 | 1:300 |
| HA | BioLegend, 16B12 | 1:3000 |
| Na^+^/K^+^-ATPase | Santa Cruz, sc-16043 | 1:500 |
| COX4 | Cell Signaling Technology | 1:1000 |
|  |  |  |
