# Supplementary figures and images for: NME3 binds to phosphatidic acid and mediates PLD6-induced mitochondrial tethering
Source: J Cell Biol. 2023 Aug 16;222(10):e202301091. doi: 10.1083/jcb.202301091 (PMC10432850; doi:10.1083/jcb.202301091)

Fig. 1D

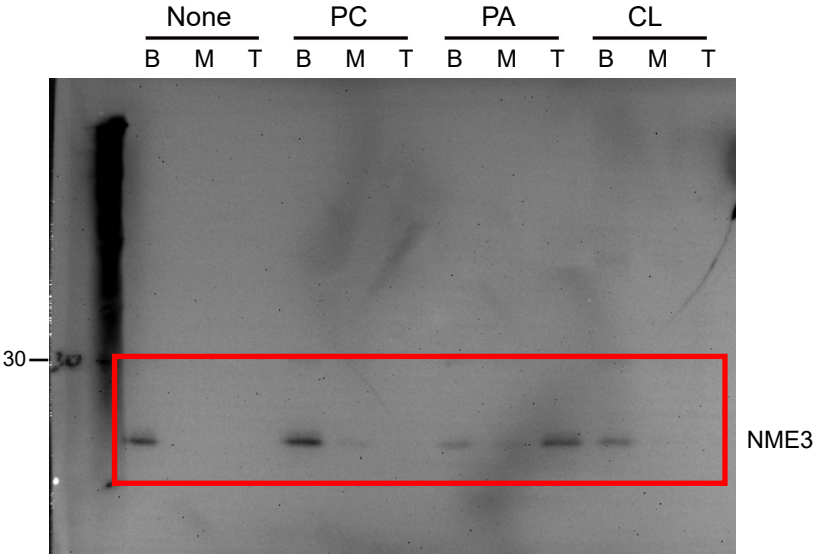

Supplement: SourceData F1 — is the source file for Fig. 1. [file JCB_202301091_SourceDataF1.pdf]

Fig. 2C

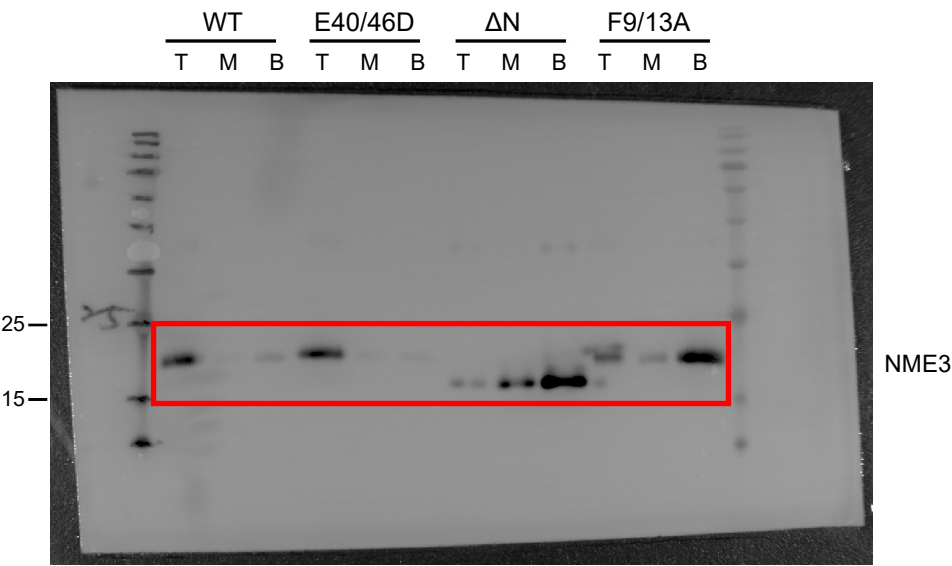

Fig. 2D

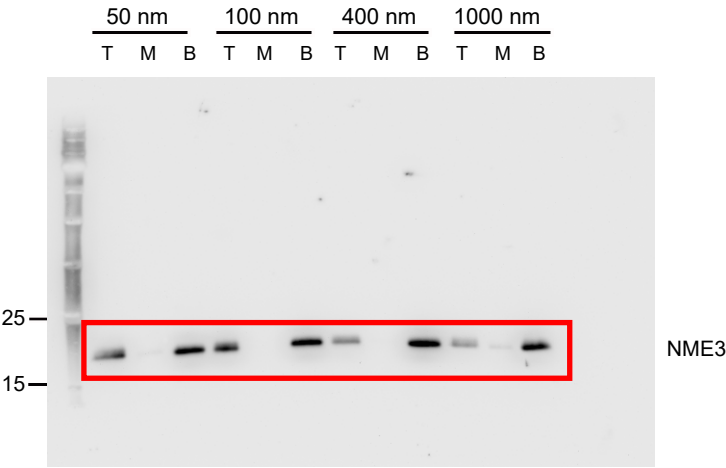

Fig. 2F

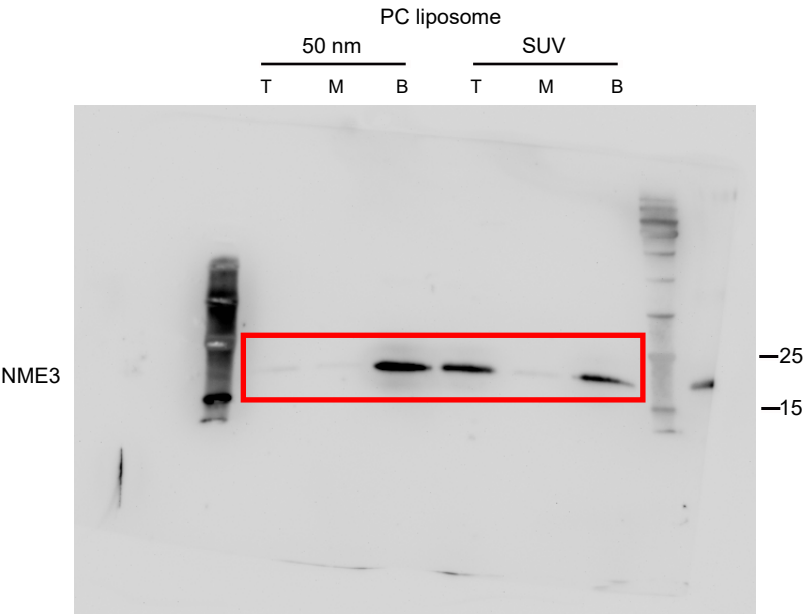

Supplement: SourceData F2 — is the source file for Fig. 2. [file JCB_202301091_SourceDataF2.pdf]

Fig. 4E

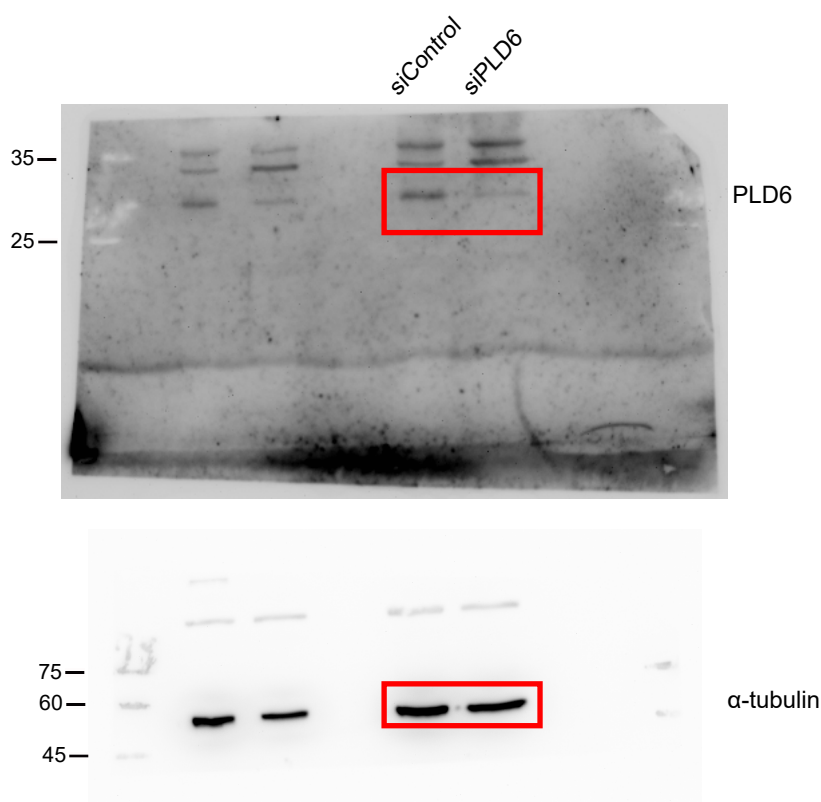

Supplement: SourceData F4 — is the source file for Fig. 4. [file JCB_202301091_SourceDataF4.pdf]

Fig. S1B

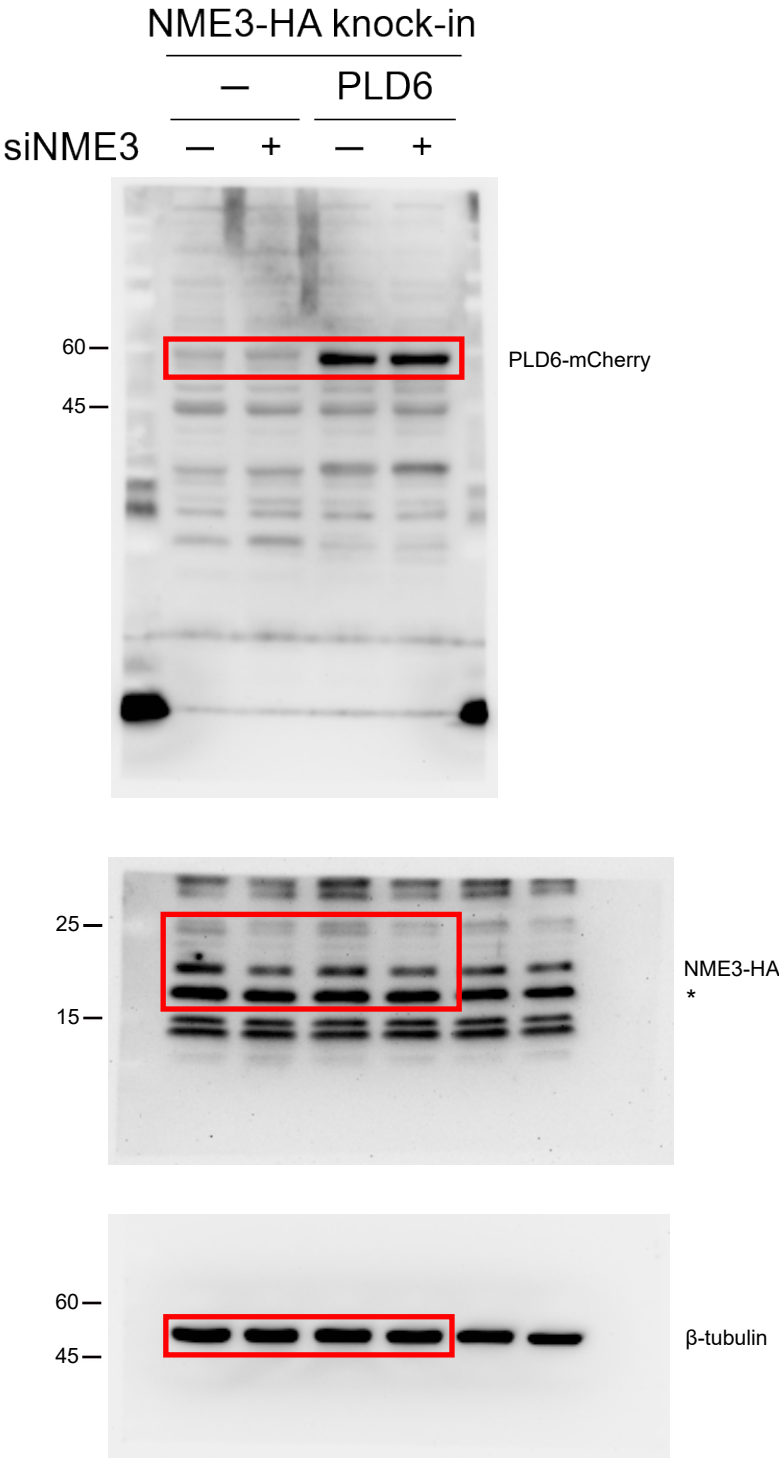

Fig. S1G

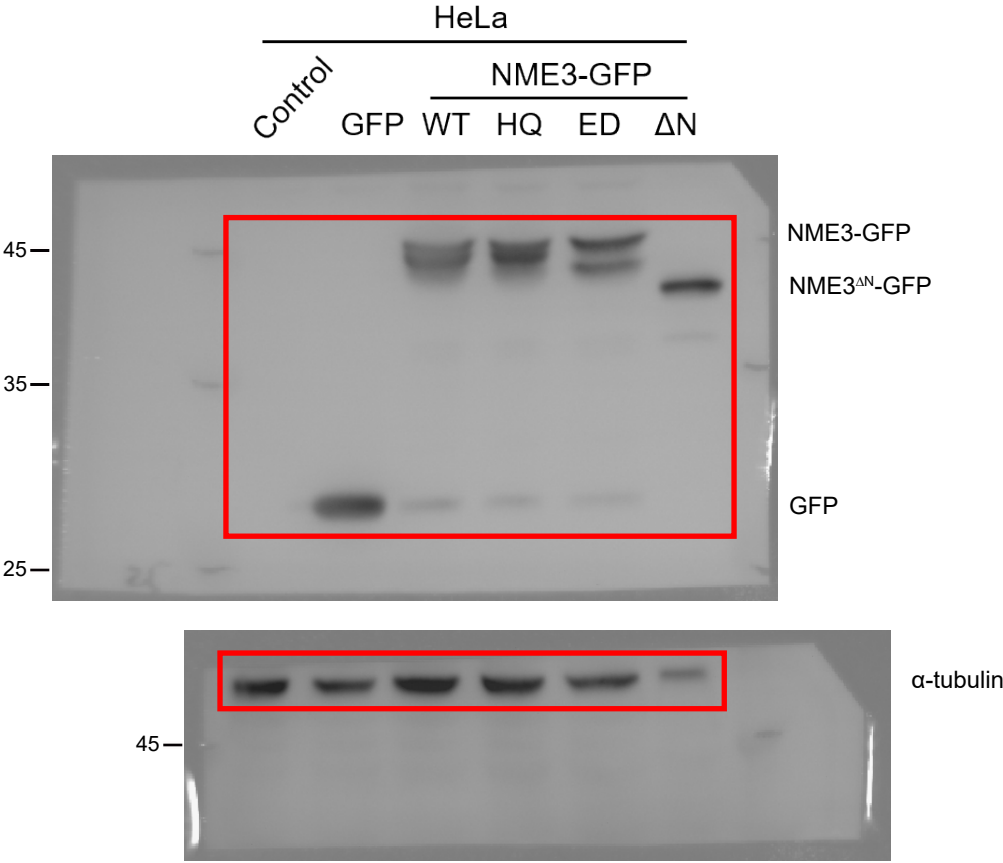

Supplement: SourceData FS1 — is the source file for Fig. S1. [file JCB_202301091_SourceDataFS1.pdf]

Fig. S2C

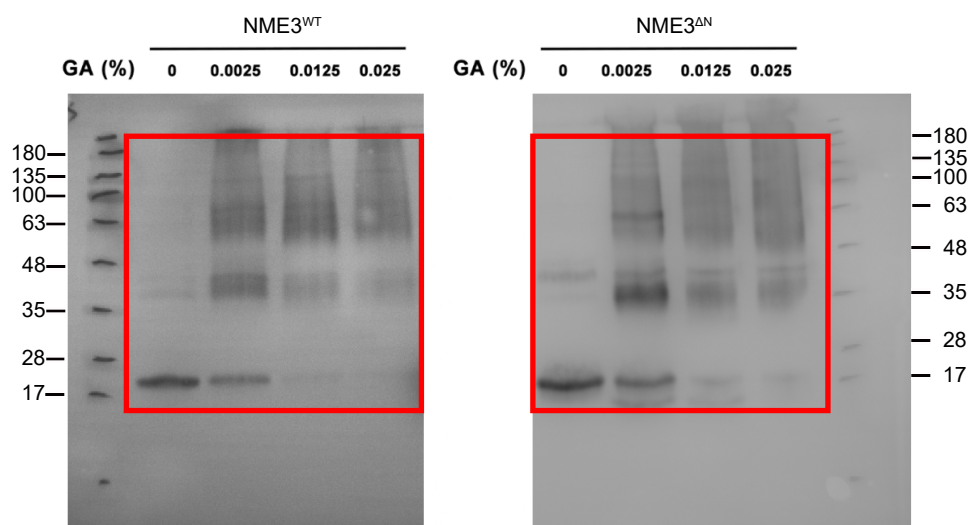

**Fig. S2D**

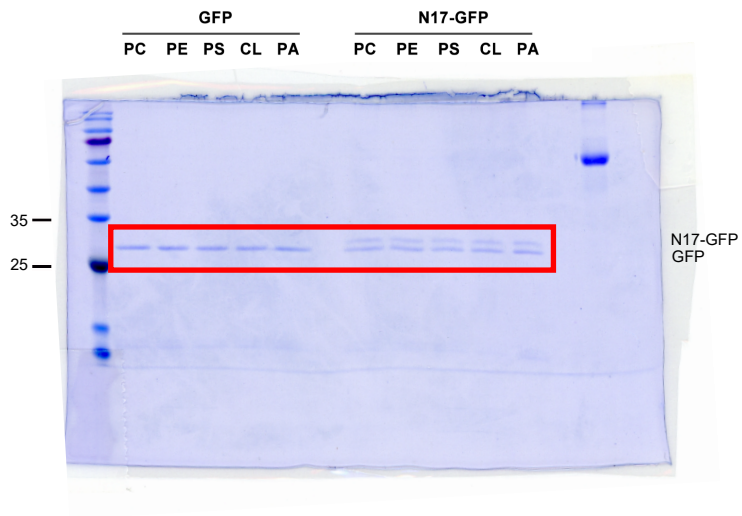

Supplement: SourceData FS2 — is the source file for Fig. S2. [file JCB_202301091_SourceDataFS2.pdf]

Fig. S3D

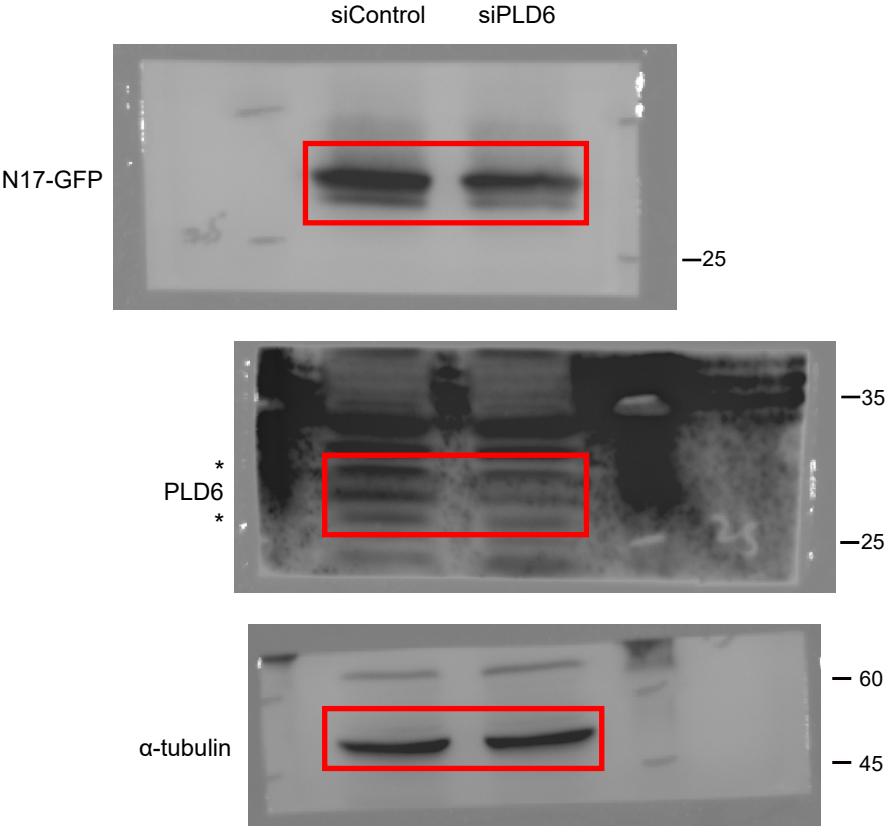

Fig. S3E

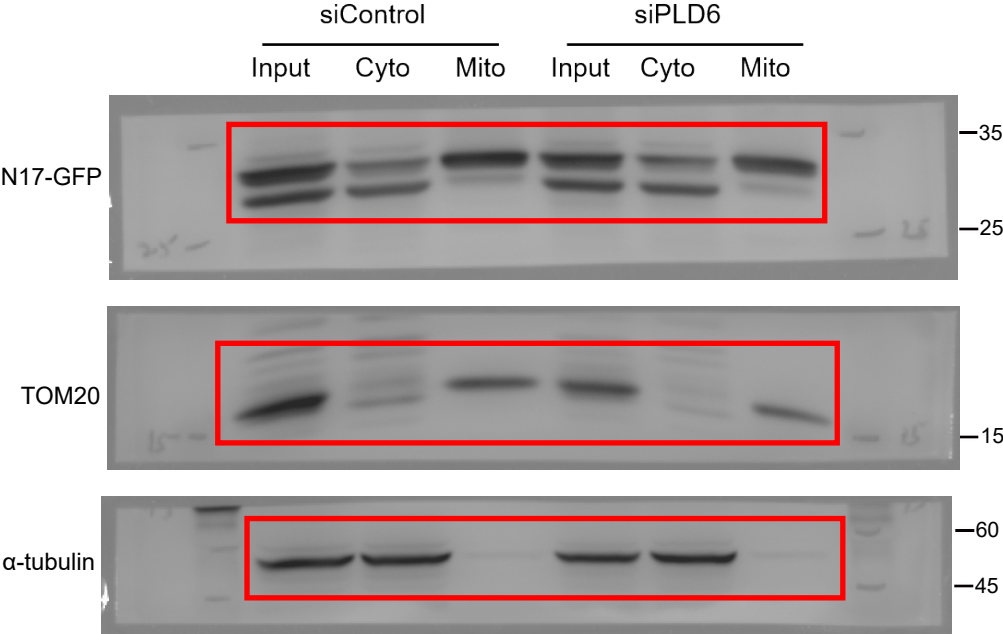

Supplement: SourceData FS3 — is the source file for Fig. S3. [file JCB_202301091_SourceDataFS3.pdf]

Fig. S4B

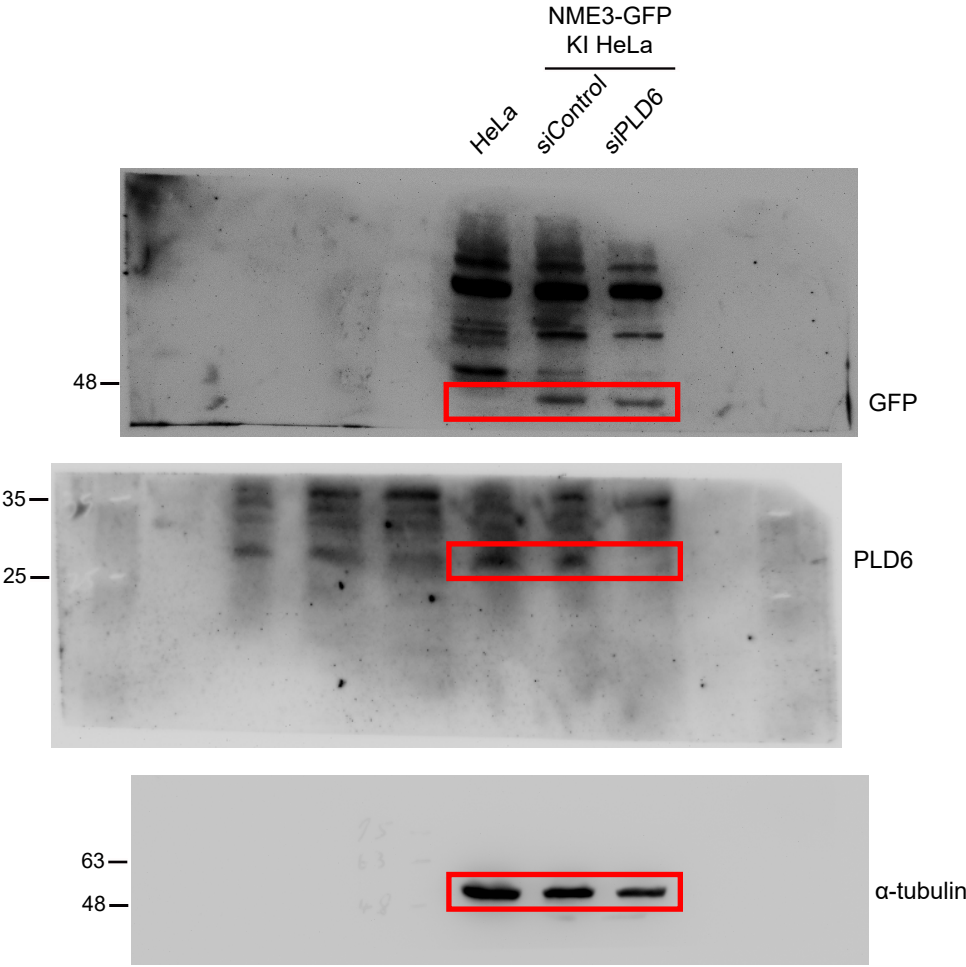

Fig. S4E

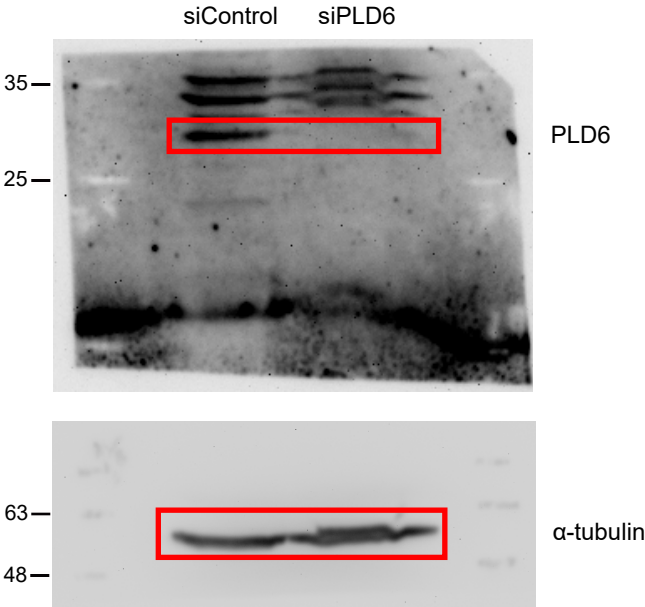

Fig. S4G

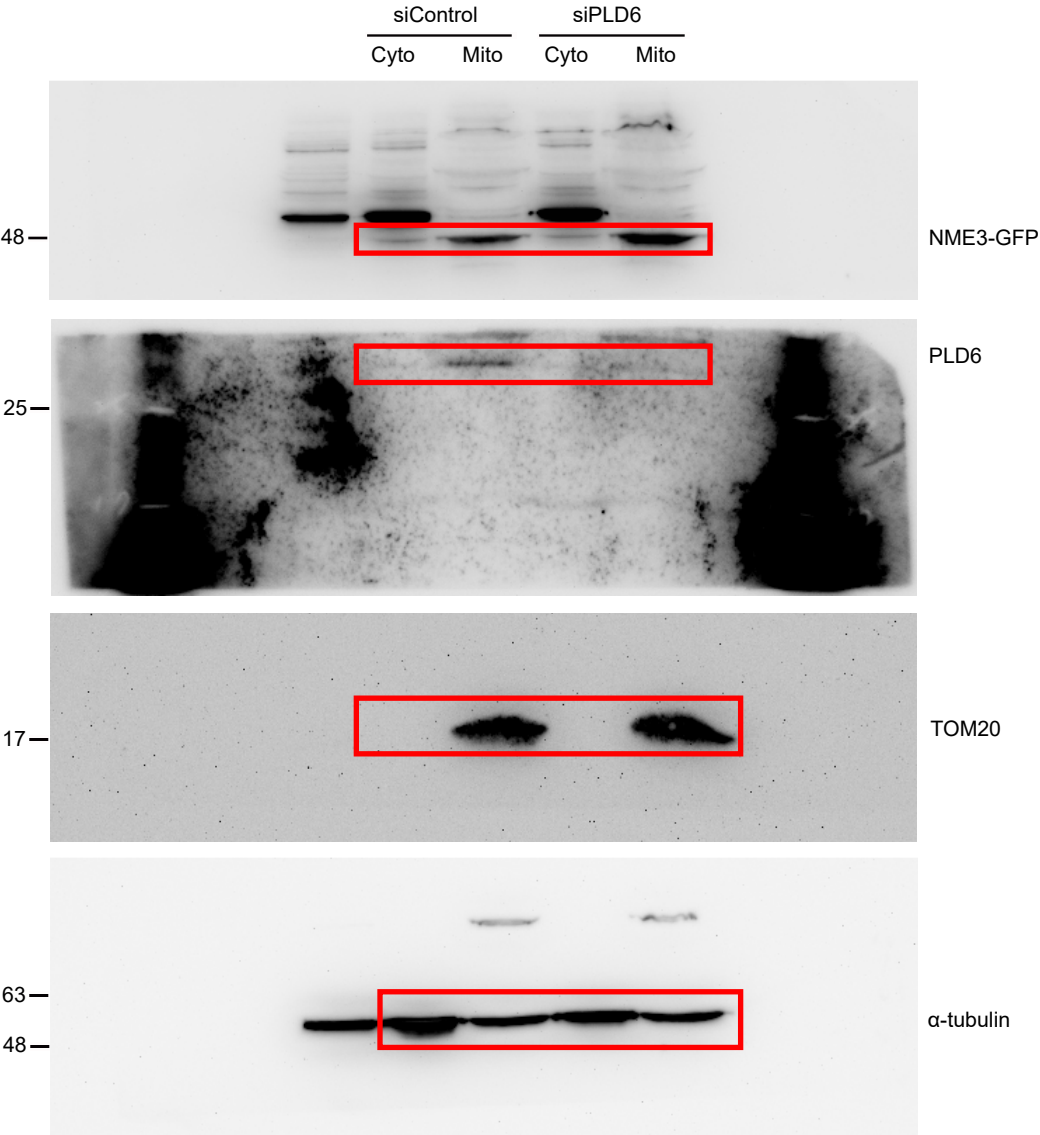

Fig. S4H

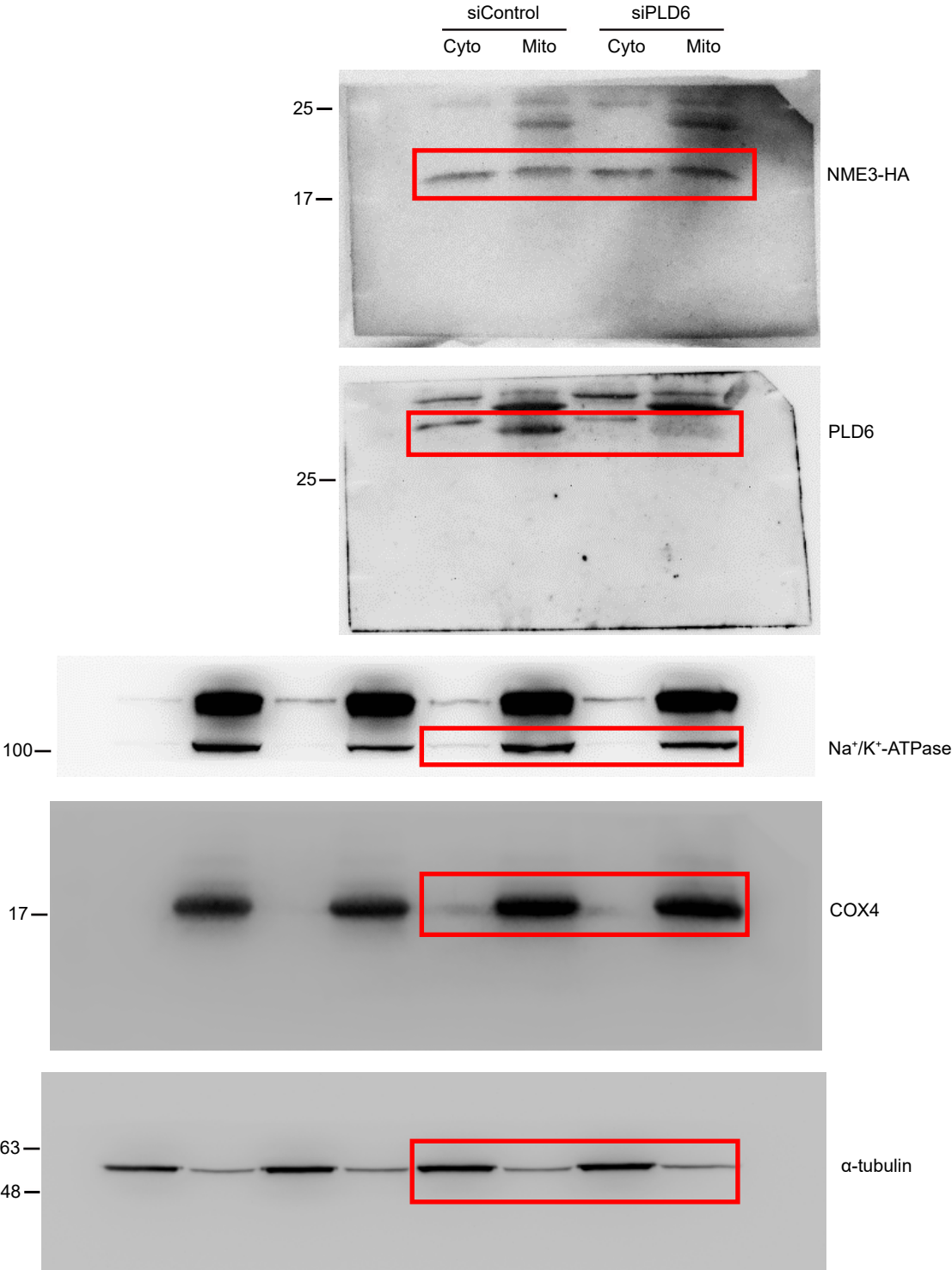

Supplement: SourceData FS4 — is the source file for Fig. S4. [file JCB_202301091_SourceDataFS4.pdf]
